# Cancer-associated S100P protein binds and inactivates p53, permits therapy-induced senescence and supports chemoresistance

**DOI:** 10.18632/oncotarget.7999

**Published:** 2016-03-09

**Authors:** Adriana Gibadulinova, Michal Pastorek, Pavel Filipcik, Peter Radvak, Lucia Csaderova, Borivoj Vojtesek, Silvia Pastorekova

**Affiliations:** ^1^ Institute of Virology, Biomedical Research Center, Slovak Academy of Sciences, Bratislava, Slovak Republic; ^2^ Regional Centre for Applied Molecular Oncology, Masaryk Memorial Cancer Institute, Brno, Czech Republic

**Keywords:** S100P calcium-binding protein, p53 tumor suppressor, HDM2, cell death, therapy-induced senescence

## Abstract

S100P belongs to the S100 family of calcium-binding proteins regulating diverse cellular processes. Certain S100 family members (S100A4 and S100B) are associated with cancer and used as biomarkers of metastatic phenotype. Also S100P is abnormally expressed in tumors and implicated in migration-invasion, survival, and response to therapy. Here we show that S100P binds the tumor suppressor protein p53 as well as its negative regulator HDM2, and that this interaction perturbs the p53-HDM2 binding and increases the p53 level. Paradoxically, the S100P-induced p53 is unable to activate its transcriptional targets *hdm2*, *p21^WAF^*, and *bax* following the DNA damage. This appears to be related to reduced phosphorylation of serine residues in both N-terminal and C-terminal regions of the p53 molecule. Furthermore, the S100P expression results in lower levels of pro-apoptotic proteins, in reduced cell death response to cytotoxic treatments, followed by stimulation of therapy-induced senescence and increased clonogenic survival. Conversely, the S100P silencing suppresses the ability of cancer cells to survive the DNA damage and form colonies. Thus, we propose that the oncogenic role of S100P involves binding and inactivation of p53, which leads to aberrant DNA damage responses linked with senescence and escape to proliferation. Thereby, the S100P protein may contribute to the outgrowth of aggressive tumor cells resistant to cytotoxic therapy and promote cancer progression.

## INTRODUCTION

S100P belongs to the S100 family of calcium-binding proteins linked with various diseases. S100P is one of the cancer-related family members implicated in cell migration-invasion and tumor metastasis [1–3]. Several studies reported the S100P association with drug resistance and protection from death in cells derived from the carcinoma of the pancreas, prostate, breast, colon etc. [4–8]. On the contrary, overexpression of S100P was shown to increase the chemosensitivity of ovarian cells [9].

These discordant effects may be caused by S100P crosstalk with diverse pathways in different tumor types, including activation of the MAPK/ERK, PI3K/AKT and NFkB signaling mediated by the binding of the extracellular S100P to RAGE receptor [10] and, based on the analogy with other S100 proteins, activation of apoptosis-related pathways driven by the JNK stress kinase and/or p53 tumor suppressor protein [1].

The wild-type p53 tumor suppressor protein plays a key role in cellular responses to DNA damage and other forms of stress, including oncogenic activation, hypoxia, and red-ox disturbance. Its expression and activation usually leads either to cell cycle arrest or to apoptosis, depending on the stress type, extent and duration, and is often associated with reduced tumor growth and good therapy response, although recent data suggest that the wild-type p53 may also contribute to treatment failure through therapy-induced senescence [11, 12]. Stability and activity of the wild-type p53 protein is determined by a number of posttranslational modifications, primarily by the phosphorylation, methylation and acetylation. These modifications also determine the interactions of p53 with its binding partners, which in turn affect the p53 modifications, stability and activity [13].

Under basal/unstressed conditions, the wild-type p53 is a short-lived protein undergoing proteasome degradation induced by its binding with the HDM2 ubiquitin ligase. Upon the DNA damage, p53 accumulates due to its phosphorylation by stress-induced kinases (such as ATM/ATR, and Chk1/Chk2) followed by the disruption of the p53-HDM2 interaction. Additional kinases then activate the ability of p53 to act as a transcription factor. This leads to cell cycle arrest and/or apoptosis [14–16]. Tumor suppressor function of the wild-type p53 is perturbed by mutations, many of which can abolish its ability to transactivate target genes [17]. Similarly, p53 activity can be perturbed by its interaction with oncoproteins (including T antigen of SV40). This interaction can stabilize and/or alter the p53 protein folding preventing either its binding to cognate promoters, tetramerization, or cooperation with transcriptional co-factors [18].

Interestingly, the cancer-related members of the S100 family (S100A2, S100A4, S100B) can interact with p53 and/or HDM2, but the modes of their interactions differ and consequences range from cancer-suppressing to cancer-promoting [19–21]. Here we investigated the interaction of S100P with p53 and HDM2 and explored the immediate and delayed effects of S100P on response of tumor cells to DNA damage. We found that S100P binds p53 and HDM2, reduces the p53 phosphorylation and affects the expression of p53 targets. We also showed that S100P facilitates the therapy-induced senescence and supports the clonogenic survival of cancer cells. These effects might explain the S100P relationship to therapy resistance and cancer progression.

## RESULTS

### S100P binds p53 and HDM2 and reduces the HDM2-p53 interaction

**Figure 1:**
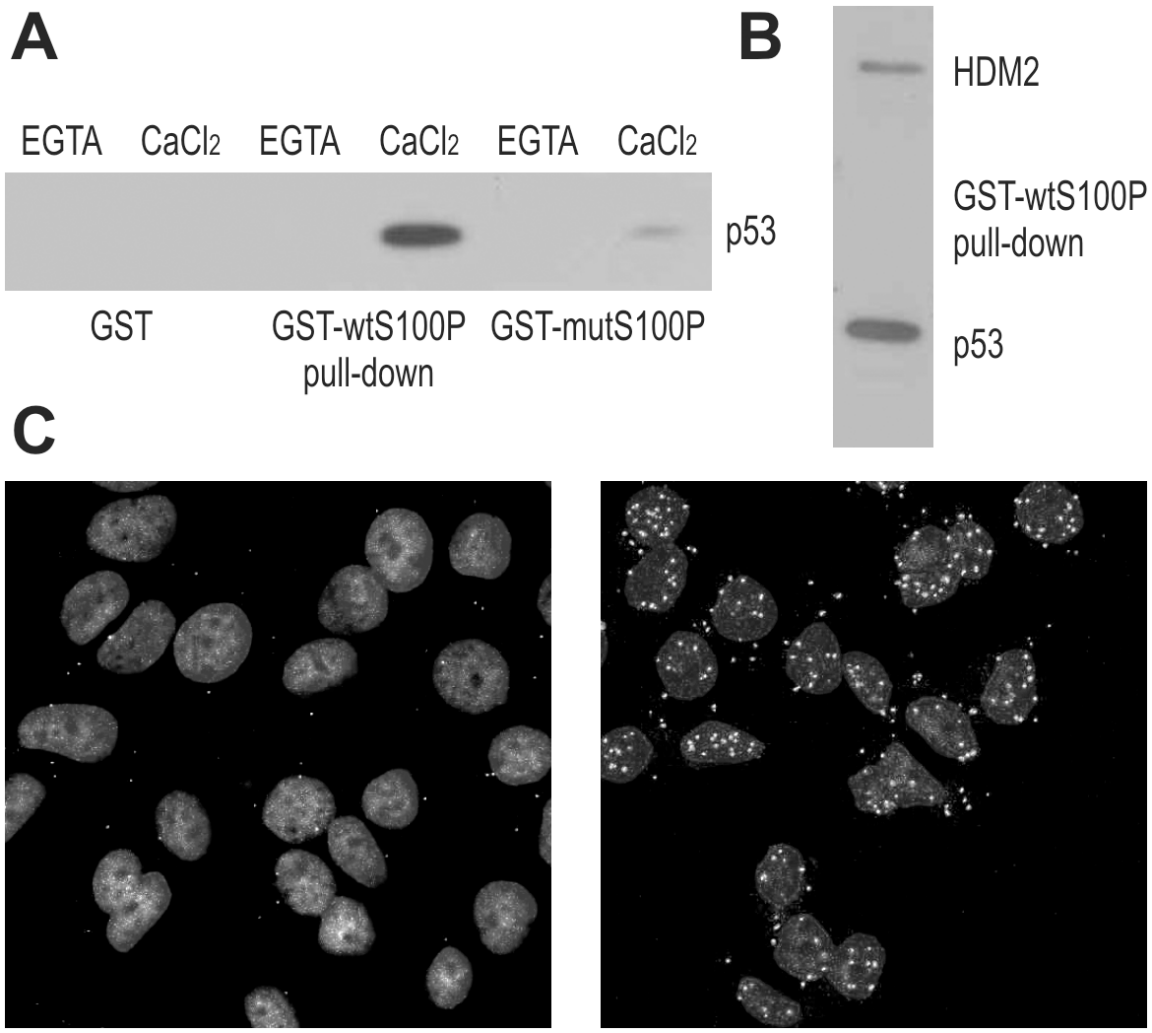
S100P Interacts with p53 and HDM2 **A.** Interaction between S100P and p53 is demonstrated by GST-pulldown from T47D cells followed by the immunoblotting with the p53-specific antibody DO-1. The blot shows that the interaction is calcium-dependent and can be diminished by the F15A mutation compromising the dimerization of S100P. **B.** GST-pulldown from the RKO cells followed by immunoblotting reveals that S100P can bind both p53 (detected by the DO-1 antibody) and HDM2 (detected by the 2A9 antibody). **C.** Proximity ligation assay of MCF7 cells with endogenous S100P expression (control in left panel and treated with dexamethasone and UV irradiation in right panel) allowed for visualization of S100P-p53 interaction *in situ*. The PLA signal represented by the white spots shows stronger and more abundant interactions in treated cells with induced expression of S100P and p53.

To study the S100P-p53 interaction, we performed pull-down experiments using the GST-wtS100P protein and the GST-mutS100P protein with the dimerization-disabling F15A mutation. The proteins were immobilized on affinity matrix and incubated with extracts of T47D cells expressing the mutated p53 and of RKO cells expressing the wild-type p53, with or without calcium ions. We found that S100P could bind both wild-type and mutant p53 in a calcium-dependent manner (Figure 1A, 1B). Moreover, the dimerization-defective S100P mutant exhibited reduced p53 binding. Interestingly, the GST-wtS100P protein could pull-down also the HDM2 oncoprotein, an ubiquitin-ligase that binds the wild-type p53 and directs it to proteasome degradation (Figure 1B). These results were corroborated by alternative approaches, including Far Western blotting of the lysates from the RKO cells expressing the wild-type p53 compared to the p53-null NCI-H1299 cells, and co-immunoprecipitation of p53 and HDM2 proteins through S100P-specific antibody from the lysates of RKO cells ectopically expressing either the wild-type or dimerization-defective S100P protein (Supplementary Figure S1).

To visualize the S100P-p53 binding in situ, we accomplished the proximity ligation assay (PLA), [22], and demonstrated that S100P interacts with the wild-type p53 in MCF-7 breast carcinoma cells (Figure 1C). Since these cells show low levels of both p53 and S100P, we first treated them with dexamethasone (10 μM) to induce S100P [23], and then with UV irradiation (12 J/m^2^) to induce p53. Expectedly, the PLA signal was rare in MCF-7 cells under basal conditions and abundant mainly in the nuclei of the treated MCF-7 cells (Figure 1C).

**Figure 2:**
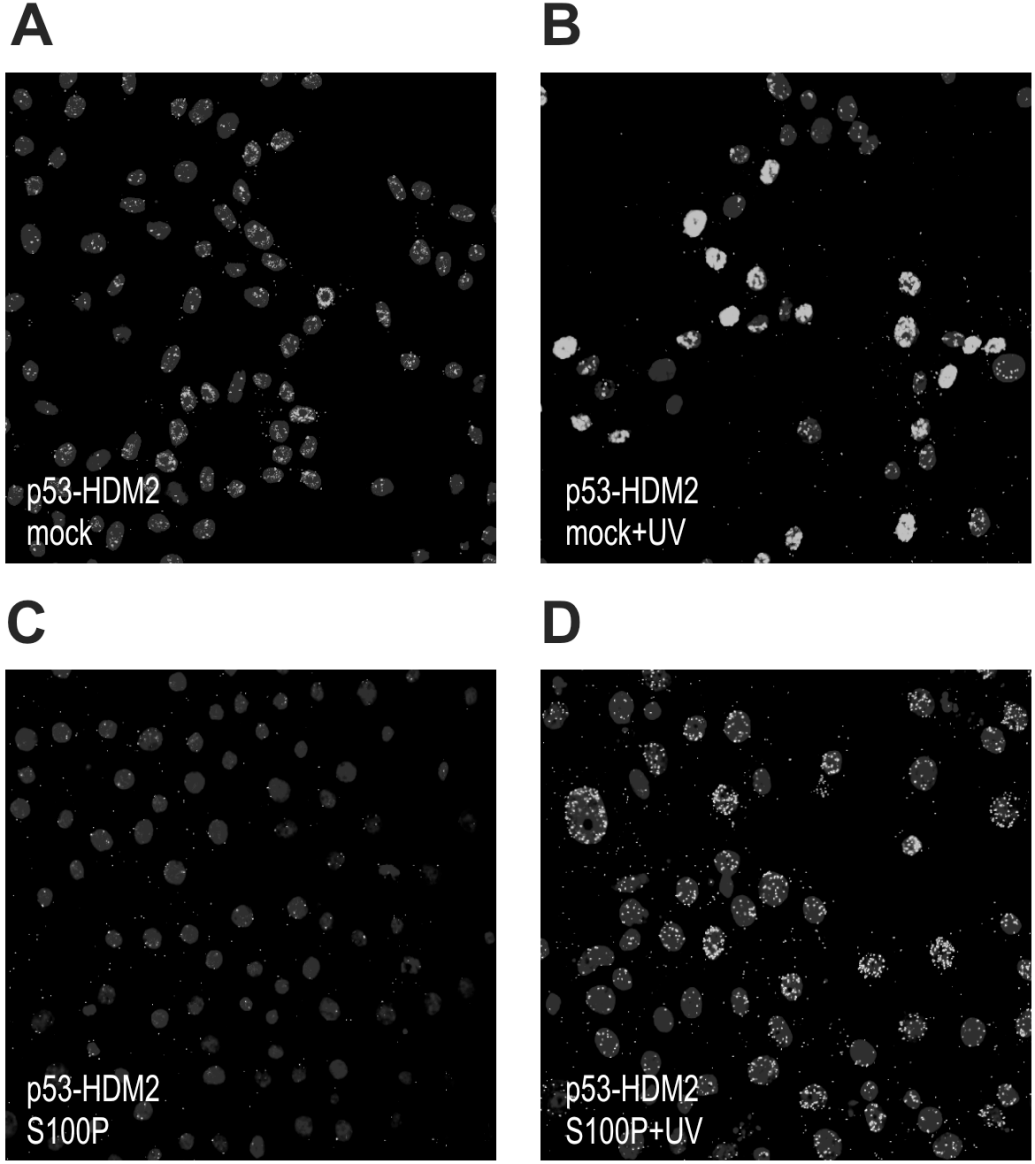
S100P perturbs the p53-HDM2 interaction The RKO cells were subjected to PLA analysis using the p53-specific rabbit polyclonal antibody CM1 and the HDM2-specific mouse monoclonal antibody 2A9. Panel **A.** shows the PLA signal for p53-HDM2 interaction in the mock-transfected cells under basal conditions, whereas panel **B.** shows the same cells after the treatment with UV irradiation, in which the signal is considerably elevated. Panels **C.** and **D.** show the S100P-transfected cells that exhibit reduced PLA signal under both basal and UV-treated conditions presumably due to S100P-perturbed interaction between p53 and HDM2.

Because the wild-type p53 protein is kept under negative control by HDM2, we wanted to learn, whether S100P interferes with the p53-HDM2 interaction. We performed the PLA with the p53- and HDM2-specific antibodies in RKO cells and in their transient S100P-transfectants. Both mock- and S100P-cells were either untreated or UV irradiated to elevate the p53 expression (Figure 2). A weak PLA signal demonstrating the wtp53-HDM2 interaction in mock-transfectants became stronger following the UV-treatment and was mainly confined to nuclei (Figure 2A, 2B). This reflected the fact that p53 and HDM2 levels increased and both proteins remained in the close proximity, consistently with the model of p53 being anchored at promoters and controlled through the adjacent HDM2 [24]. In the presence of ectopic S100P, the PLA signal became less prominent and was also outside of nuclei suggesting that the S100P binding to p53 and HDM2 perturbed their mutual interaction and stimulated their nuclear export (Figure 2C, 2D).

### S100P increases the level but not the activity of the wild-type p53

**Figure 3:**
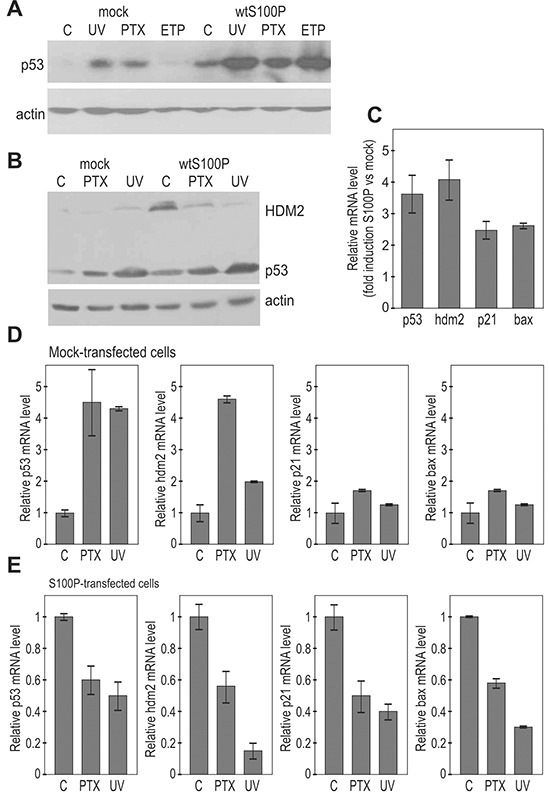
S100P affects the expression of p53 and its transcriptional targets **A.** Immunoblotting analysis of the p53 protein levels in relationship to S100P expression in A549 cells. The transiently transfected cells expressing S100P showed higher p53 protein level in both basal and treated conditions. **B.** Immunoblotting analysis of p53 and HDM2 protein levels in the mock-transfected and S100P-transfected RKO cells. RKO-S100P cells express higher levels of p53 and HDM2, but the level of HDM2 was not increased in response to treatment. UV = UV irradiation, PTX = paclitaxel, ETP = etoposide. **C.** Q-PCR analysis of p53 and its targets in the non-treated S100P vs mock-transfected cells shows increased levels of transcripts in the presence of S100P, **D.** Transcriptional analysis of p53 and its target genes in the mock-transfected RKO cells. Data show induction of the p53 mRNA itself as well as of the p53 protein targets in response to treatments. **E.** Transcriptional analysis by Q-PCR in S100P-expressing cells showed reduced levels of the analyzed transcripts in the cells subjected to treatments.

Next we asked whether the S100P-p53 interaction could affect the p53 expression and/or function. Thus, we analyzed the p53 protein levels in A549 and RKO cells, which normally express low levels of the wild-type p53, and display either moderate expression (A549) or absence of S100P (RKO), [25]. We examined both mock-transfected and S100P-transfected cells under non-stressed conditions and following the DNA damaging treatments, including UV-irradiation, paclitaxel (PTX) and etoposide (ETP). Both A549 and RKO mock-transfected cells showed low basal levels of p53, which were elevated following the treatments. However, the basal as well as induced levels of the p53 protein were elevated in the presence of S100P (Figure 3A, 3B). Such increase is clearly visible also in MCF-7 cells with endogenous S100P expression (Supplementary Figure S2A). This might be related to the reduced p53-HDM2 interaction leading to decreased p53 degradation. Also HDM2 level was higher in the S100P-RKO cells compared to controls, in accord with the known p53-HDM2 feedback loop, through which p53 induces its own negative control [24]. However, no further induction of HDM2 but its decrease was observed following the treatments, indicating that the S100P-elevated p53 might become less active (Figure 3B).

Indeed, the Q-PCR analysis revealed that under basal conditions the S100P expression led to an increased transcription of the p53 mRNA itself and of its transcriptional targets *hdm2*, *p21^WAF^*, and *bax* (Figure 3C). However, the DNA damage-inducing treatments increased the levels of the analyzed mRNAs only in the absence of S100P (Figures 3D, 3E). The presence of S100P in RKO cells led rather to the decreased levels of all analyzed mRNAs in response to treatments supporting the view that S100P-mediated elevation of the p53 expression is connected with the inactivation of the p53 protein in terms of its transactivation ability. Similar results were obtained in A549 cells (data not shown).

### S100P affects p53 phosphorylation and modulates expression of cell death-related proteins

In order to disclose S100P-induced molecular changes, we analyzed the expression pattern of a collection of cell death-related proteins, some of which are linked with the tumor-suppressor function of the wild-type p53. We used the human apoptotic proteome profiler array. The membranes with an array of antibodies were incubated with the cell lysates of the transiently mock- and S100P-transfected RKO cells, non-treated or subjected to treatment with paclitaxel, etoposide and camptothecin, respectively. The treatment was allowed to proceed for the relatively short time periods (4-6 h) and thus the observed changes could be attributed to initial cell responses to the DNA damage.

**Figure 4:**
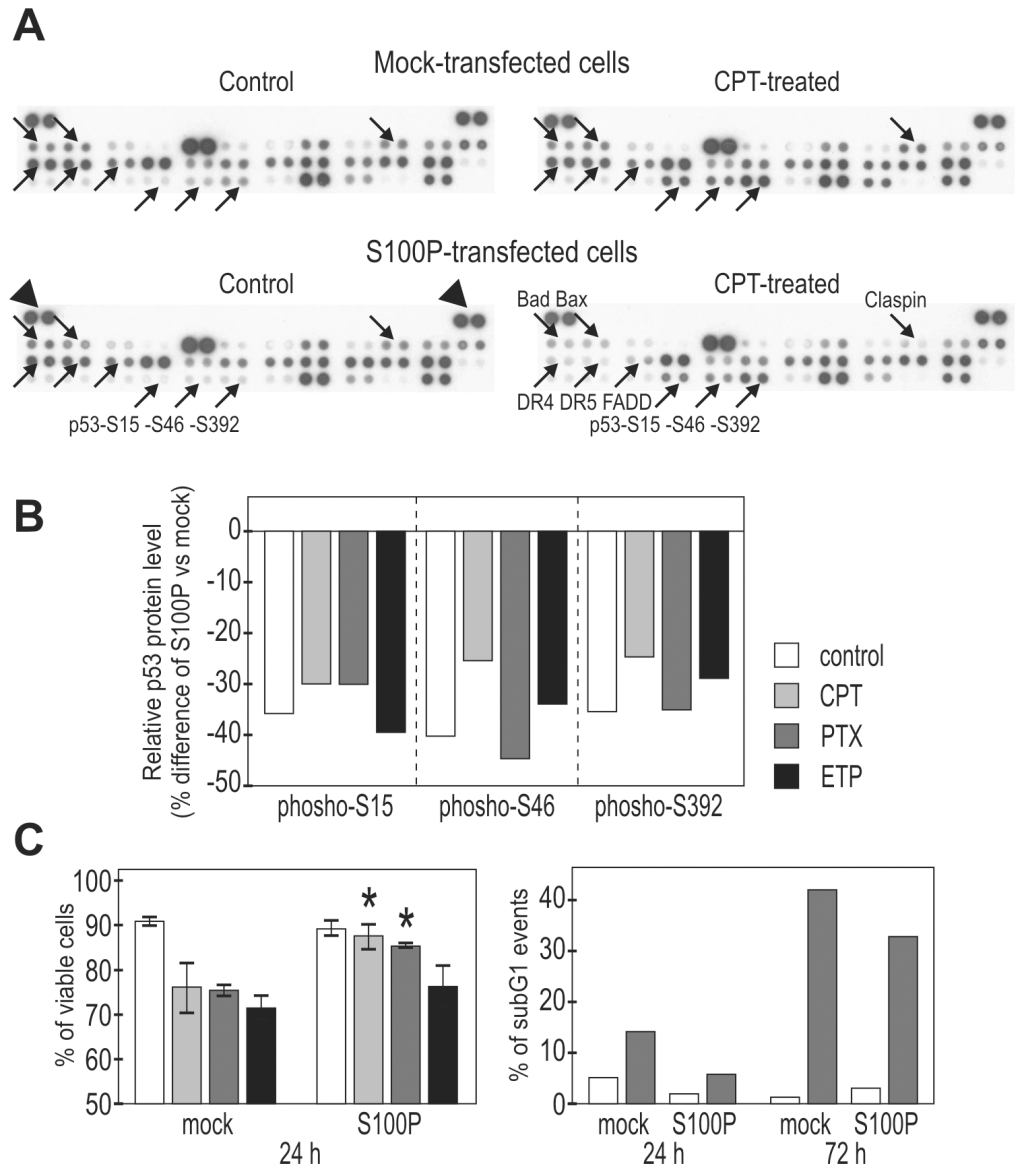
S100P influences the expression of cell death-associated proteins and improves cell viability **A.** Protein expression was analyzed using the proteome-profiler array in extracts from the mock-transfected, camptothecin-treated (6h) vs untreated cells and in the transiently S100P-transfected, treated vs. untreated cells. Proteins showing remarkable changes are indicated by arrows and named at one of four corresponding panels. **B.** Graphical illustration of the changes in the p53 phosphorylation. All S100P expressing cells consistently showed decreased levels of phospho-serines upon treatment with different drugs (PTX=paclitaxel, ETP=etoposide, CPT=camptothecin). **C.** Graphical illustration of the cell viability following the drug treatment (determined by the propidium iodide and fluorescein diacetate staining of intact (non-fixed cells), left panel, and by the DNA labeling with propidium iodide in fixed cells, right panel). S100P-expressing cells (stable transfected mixed populations) showed significantly (*) increased viability compared to mock-transfected controls.

We found clear differences between the mock-transfected and transiently S100P-transfected RKO cells both under basal and drug-treated conditions, as exemplified on the profile of the camptothecin-treated cells (Figure 4A). The most prominent changes were related to the phosphorylation of three serine residues of p53, which was consistently reduced by 30-50% in the S100P-expressing cells (Figure 4B). This was in agreement with the above-proposed S100P-mediated inactivation of p53 function, since particularly the phosphorylated N-terminal Ser15 and Ser46 appear to affect the p53 transactivation potential [14, 26]. We also observed reduced levels of pro-apoptotic proteins including Bad, Bax, DR4, DR5 and FADD (Figure 4A), suggesting that the S100P expression led to attenuated cellular response to the cytotoxic insult. This finding was supported by the FACS analysis at later time points (24 and 72 h post-treatment with PTX), which showed reduced cell death in the presence of S100P (Figure 4C).

### S100P influences cellular responses to DNA damaging drugs and supports therapy-induced senescence

**Figure 5:**
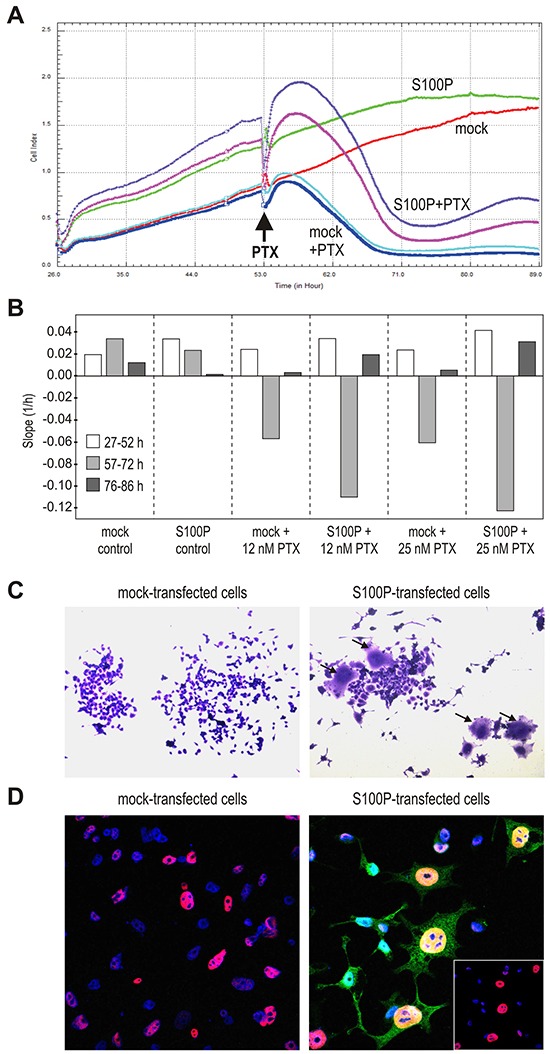
S100P induces the senescence-like morphology **A.** Impedance-based real-time measurement of cell proliferation and/or death. Impedance values from quadruplicates are expressed as Cell index. **B.** Slopes derived from the same measurement data indicate the speed of changes in the cell numbers and/or cell-covered areas. **C.** Morphology of cells 72 h post-treatment with PTX, with the subset of S100P-expressing cells showing the senescence-like phenotype with flattened, granular appearance and visibly enlarged size (arrows). **D.** Immunostaining of p53 (red) and S100P (green), combined with the nuclear staining (blue) 72 h post-treatment with PTX. S100P and p53-positive cells display typical senescent morphology and contain abnormally large nuclei. Bottom right inlet reveals the p53 expression in the DAPI-stained nuclei after suppression of the S100P signal from the confocal image.

In order to better understand biological effects of S100P, we evaluated cell proliferation and cytotoxic responses in the real-time setting using the xCELLigence system, which measures the electrical impedance across the gold microelectrodes integrated in the bottom of microplates. There, the attachment, spreading and growth of cells resulting in an increased coverage of the bottom area increase the impedance, whereas detachment and cell death cause its reduction. We evaluated the RKO-mock cells versus transiently transfected RKO-S100P cells either in control conditions or after the treatment with 5 nM or 25 nM PTX (Figure 5A).

Both S100P-positive and negative non-treated RKO cells displayed continuous proliferation, which appeared to be faster for the S100P transfectants especially in the first phase (at 27-52 h post-plating). Subsequently, growth of the RKO-S100P cells was slightly slower (at 57-72 h post-plating) until reaching the plateau (at 76-86 h), (Figure 5B). The mock controls grew initially a little slower, then slightly speeding and reaching the plateau somewhat later than S100P-positive cells. After the addition of PTX, both mock- and S100P-transfected cells started to detach and/or die out. However, while the PTX-treated RKO-mock cells reached the bottom value and remained there during the entire third phase of the recording (at 76-86 h post-plating), the cell index of the RKO-S100P cells first fell down and then modestly increased (Figure 5A, 5B). Similar profile was seen with the S100P-transfected A549 cells.

To understand this finding, we inspected the appearance of the RKO cells treated with CPT for 72 h and then allowed to restore in fresh medium for additional 72 h, fixed and stained. Interestingly, the S100P-transfectants that survived the drug treatment contained rare cells with a senescence-like morphology characterized by spread, flattened shape with an increased cytoplasmic granularity (Figure 5C). These cells covering an enlarged bottom area could be at least partially a reason for the increased impedance observed above.

We then wanted to know, whether these flattened cells express S100P and/or p53. Thus, we triple-stained the cells surviving the drug treatment with antibodies against S100P and p53 and with DAPI to visualize the nuclei. Confocal microscopic analysis showed the nuclear p53 staining in both mock- and S100P-transfected cells, but the p53-positive nuclei of the subset of S100P-expressing cells were much larger and had an aneuploid-like appearance, which is another feature of senescent cells (Figure 5D). These data indicated that the cells that express simultaneously S100P and p53 are able to withstand the cytotoxic treatment and appear to acquire the senescent morphology.

### S100P-mediated therapy-induced senescence is linked with clonogenic survival

**Figure 6:**
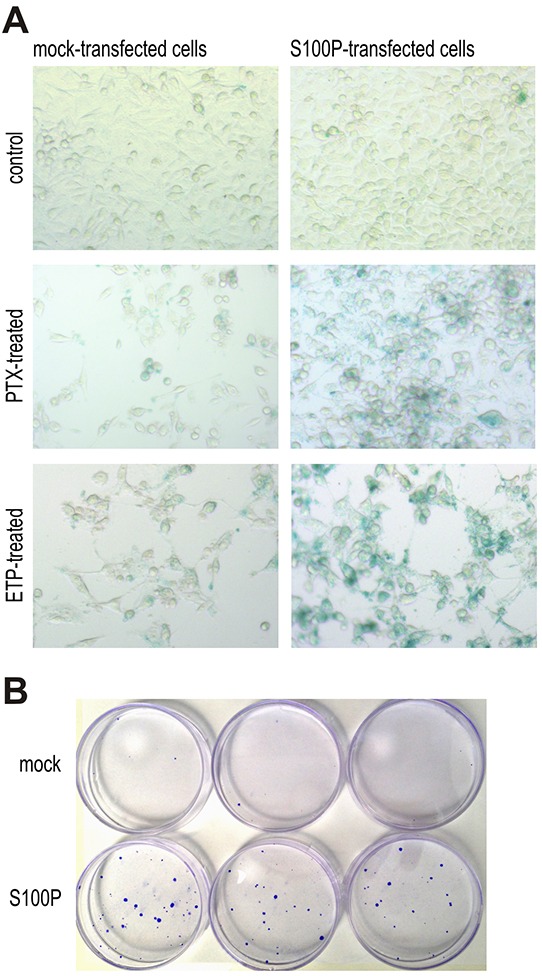
S100P contributes to therapy-induced senescence and survival **A.** Detection of senescence by SA-β-galactosidase assay. Blue senescent cells were more frequent in PTX and ETP-treated S100P expressing RKO cells compared to mock controls, whereas no difference between these cell variants is visible under basal non-treated conditions. **B.** Representative image of colonies formed from the S100P-overexpressing RKO cells and mock control cells surviving the CPT treatment.

To verify the assumption that S100P can support the onset of therapy-induced senescence, we performed the SA-β-gal assay. The blue color resulting from the increased lysosomal activity of the senescent cells was virtually not present in the non-treated cells. However, treatment with the PTX and ETP induced a strong SA-β-gal staining in the S100P-transfectants, whereas the mock-transfected cells showed only a faint signal suggesting the S100P involvement in the therapy-induced senescence (Figure 6A).

Cellular senescence induced by therapy is currently perceived as one of the mechanisms protecting tumor cells from death and allowing them to temporarily resist cytotoxic drugs [27–30]. This can lead to prolonged survival, selection and outgrowth of resistant cell subpopulation potentially causing therapy failure and cancer progression. To find out, whether the S100P expression can contribute to therapy resistance, we performed a colony outgrowth assay, which showed that the treatment with CPT and PTX, respectively, followed by the prolonged incubation almost completely eliminated the mock-control RKO cells, whereas few S100P-transfectants remained viable and established small, but visible colonies (Figure 6B). Average number of colonies formed from 1000 plated cells/dish after CPT treatment was 1.5±0.86 for the mock-transfected cells and 19.0±1.73 for the S100P-transfected cells (p=0,000097), and after PTX treatment 2.83±2.75 for the mock controls versus 20.2±3.7 for the S100P transfectants (p=0.00043).

**Figure 7:**
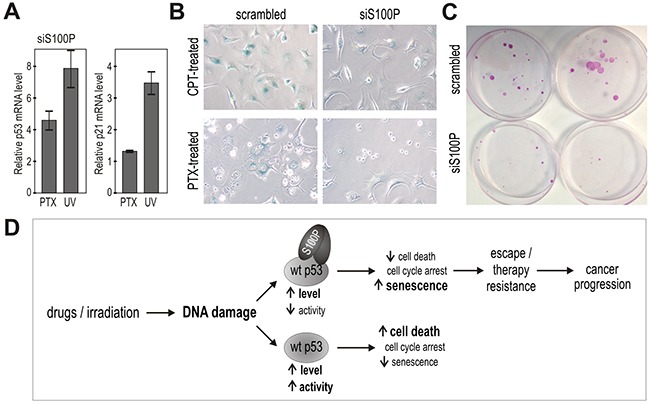
Suppression of endogenous S100P reduces senescence and survival, and supports the proposed mechanism of the S100P contribution to cancer progression **A.** Q-PCR analysis of the S100P-silenced versus scrambled MCF-7 cells showed increased levels of the p53 and p21^WAF^ transcripts in response to treatment with PTV and UV, respectively, in contrast to the results in the treated S100P-overexpressing versus control RKO cells as shown above on Figure 3E. **B.** S100P-silenced MCF-7 cells also showed weaker SA-β-gal staining compared to scrambled control. **C.** Representative image of colonies formed from the S100P-deficient MCF-7 cells and scrambled control cells surviving the PTX treatment. **D.** Schematic illustration of the proposed mechanism of S100P action in cancer cells exposed to DNA damage. In the wild-type p53-competent tumor cells, cytotoxic treatment triggers the DNA damage response that induces the accumulation of the p53 protein and its increased transactivation activity, which then leads preferably to cell death. In the presence of S100P, the wild-type p53 levels increase as a result of the S100P-p53 interaction, but the transactivation capacity is lowered. This suboptimal p53 activation is apparently not sufficient to induce full death response and the cells tend to enter the senescence program as evidenced by their morphology and expression profile. Such S100P-promoted shift in the phenotype of the subset of tumor cells with the wild type p53 can potentially lead to cancer progression through senescence escape and therapy resistance.

Moreover, we accomplished knockdown experiments leading either to transient or stable S100P silencing in MCF-7 breast carcinoma cells that display endogenous S100P expression. Despite the fact that the level of the endogenous S100P protein is lower compared to the ectopic S100P level in the transfected cells, the effects of silencing versus scrambled control could be seen with respect to an increased p53 transcription and p21 transactivation (Figure 7A), reduced SA-β-gal staining (Figure 7B) and loss of ability to survive the treatment with PTX and form large colonies (Figure 7C), with the average number of colonies formed from 1000 plated cells/dish corresponding to 7±2 for the S100P-deficient cells versus 22.3±2.31 for the S100P-compentent MCF7 cells (p=0.00029). Interestingly, a long-term (over 3 months) incubation of the MCF-7 cells in the presence of increasing concentrations of PTX led to the selection of PTX-resistant cell line, which showed increased expression of S100P apparently due to the enrichment of the S100P-positive cells (Supplementary Figure S2). These data support the view that S100P actively participates in an acquisition of the resistant tumor phenotype.

## DISCUSSION

This study aimed at better understanding of the role of S100P protein in the response of tumor cells to cytotoxic therapy. This issue has remained controversial, since certain studies claim the S100P involvement in therapy resistance, whereas the others suggest its role in chemosensitivity [1]. These dichotomous outcomes may be related to different cell models, drugs, and clinical samples. Also the timing of experiments can matter, since the onset of quiescence is usually fast, followed by death-response, whereas adaptive/protective mechanisms, including senescence and senescence-escape, require a longer time-frame [11]. The situation is complicated also because the S100P protein can elicit its effects either through the extracellular stimulation of the RAGE receptor activating MAPK, PI3K and NF-kB pathways [10], or through the intracellular modulation of proteins interacting with S100P, e.g. the chaperone-associated proteins HOP and CHIP that affect proteasome degradation of many proteins, including p53 [31].

We decided to look closer at this phenomenon in conjunction with the p53-related responses. We were inspired by the fact that cancer-related S100 family members interact with p53 and modulate its DNA binding, oligomerization and/or transactivation activity [32–34].

Interestingly, the modes of the p53 binding by the S100 proteins and impacts on the p53 activity are not identical, albeit all seem to be calcium-dependent. Binding of S100 proteins to the tetramerization domain (TET) of p53 seems to be a general property of the family, whereas binding to the C-terminal negative regulatory domain (NRD) is not common [33]. Some S100 proteins can also bind to p53 through its N-terminal transactivation domain (TAD) [34]. Binding of the S100A2/-A6/-A9 to the C-terminal region of p53 leads to its increased transactivation ability presumably resulting in increased death of cancer cells [20, 35, 36]. The S100B interaction with p53 involves both NRD and TET, inhibits the PKC-mediated phosphorylation and promotes disassembly of the p53 tetramers [21, 37]. This leads to the decreased DNA binding and reduced transactivation by p53. Thus, it was proposed that the elevated S100B in cancer inhibits p53 functions and supports cancer progression [21]. The S100A4 (known as MTS1 metastasis-related protein) binds to the C-terminal part of p53, causing its sequestration, and downregulation of p21^WAF^ and upregulation of Bax. This seems to result in elimination of cells expressing the wild-type p53 and selection of cells with aggressive phenotype [19, 38].

Here we showed that also S100P protein binds p53 in a calcium- and dimerization-dependent manner and that the binding reduces the p53 phosphorylation at Ser15 and Ser46 in the TAD and Ser392 in the NRD. This suggests that the S100P hinders the access of relevant kinases to their substrate serine residues. In addition, the N-terminal region of p53 mediates interaction with an oncogenic ubiquitin-ligase HDM2, which maintains the p53 level low by the proteasome degradation. Interestingly, S100P also binds HDM2 and compromises the p53-HDM2 interaction as evidenced by our PLA and co-immunoprecipitation data. Therefore it is not surprising that the presence of S100P is linked with the elevated p53 protein that presumably escapes recognition by HDM2 and degradation in the proteasome. This is in agreement with the data obtained with other S100 proteins that can also bind HDM2 but do not form ternary complex with HDM2 and p53 [39].

Although the S100P interaction with p53 results in its elevated expression, it is linked with the decreased activation of the p53 transcriptional targets in response to DNA damage. Based on these data we believe that S100P reduces the wild-type p53 transactivation activity through the mechanisms that may involve the S100P-p53 binding and either the steric inhibition of the p53 phosphorylation or, based on the analogy with the related S100 proteins, inhibition of the p53 oligomerization. Both phosphorylation and oligomerization were shown to be required for the p53-mediated responses to the DNA damaging treatments, although the extent of their involvement and the threshold required for the full p53 activity appear to be cell type- and cell context-dependent [26].

The p53-mediated transactivation is known to have a profound impact on molecular and cellular responses of cancer cells to cytotoxic drugs, generally inducing cell cycle arrest or cell death, and suppressing senescence, with the outcome depending on the level/extent of p53 activation, and on the severity/duration of stress. Actually, DNA damaging drugs used at concentrations that do not induce p53 to levels and activities sufficient for death, can permit the therapy-induced senescence [11]. In addition, the p53-driven responses have also temporal aspects, as cell cycle arrest and death can be triggered relatively early after a cytotoxic insult (from hours to 2-3 days) but senescence is delayed (beyond 5 days).

Because the S100P protein reduces the p53 transactivation activity, we expected that it could interfere with these cellular processes. Interestingly, the S100P-expressing, drug-treated RKO cells differed from the mock-transfected cells by the reduced expression of several important pro-apoptotic proteins, including the p53 target Bax, thus indicating a down-regulation of the death-related signaling. This down-regulation was observed shortly after the drug addition (coincidently with reduced p53 phosphorylation) and was also reflected by the increased viability of the S100P-expressing cells during the first two-to-three post-treatment days. During that period, cell numbers declined as indicated by the lowered impedance values, FACS data, values, FACS and appearance of cell monolayers (see Figures 5 and 6). However, later on, cells expressing S100P (either ectopically or endogenously) showed the ability to survive the drug treatment and form colonies, in which rare cells acquired the senescent phenotype.

The therapy-induced senescence is an important phenomenon, which can be triggered in tumor cells with the compromised function of tumor-suppressor proteins after exposure to anticancer agents and ionizing radiation [27–30, 40]. This phenomenon can protect the subset of tumor cells from therapy and promote malignant progression through adverse effects, including the production of cytokines mediating paracrine signaling and inflammation, the ECM remodeling, and EMT [41, 42]. We propose that the oncogenic potential of S100P can be connected with its ability to bind and reduce the p53-dependent cell-death response to cytotoxic treatment, and to induce MAPK/ERK as well as PI3K/AKT growth-promoting pathways which are involved in therapy-induced senescence [43,44]. Although this intracellular mode of S100P action represents just one of many facets of the S100P function in cancer biology, it may become clinically relevant particularly in tumors, which progress through disabling the wild-type p53 function.

We also cannot exclude the extracellular action of S100P, which can bind its RAGE receptor and activate major regulatory pathways [10, 31]. These responses appear to involve an internalization of RAGE [45]. Interestingly, RAGE has recently been associated with the restored adipogenesis of senescent preadipocytes through direct binding and inhibition of the cytosolic p53, a situation theoretically corresponding to the senescence escape by tumor cells [46]. Although these RAGE-related data were obtained using non-cancer models, it is conceivable that the S100P-induced effects leading to senescence and therapy resistance observed in our study might be at least partially mediated by the extracellular fraction of S100P secreted from the S100P-expressing cells.

Additional mechanism potentially contributing to the observed effects of S100P may include interaction with HDM2, which *per se* is an oncoprotein that can regulate cell proliferation and survival also in the p53-independent manner through transcriptional regulation of multiple target genes, chromatin remodeling and control of mRNA stability and translation [47, 48]. However, understanding a possible role of S100P in this complicated network of the p53-independent HDM2 activities is beyond the scope of this work.

In conclusion, we showed for the first time that: (a) S100P binds p53 protein and increases its level, (b) this binding leads to reduced p53 phosphorylation and transactivation activity in response to DNA damaging treatments, (c) through the inactivation of p53, S100P permits the onset of therapy-induced senescence and supports survival of the drug-treated tumor cells (see the scheme on Figure 7D). Such mode of action is compatible with the data relating S100P expression to therapy resistance and classifies S100P among the pro-metastatic members of the S100 family, such as S100B and S100A4 [1]. Our findings thus provide a new insight into the molecular mechanisms used by S100P to facilitate cancer progression and suggest that it may become a promising target for the wild-type p53 activity-preserving anticancer strategies.

## MATERIALS AND METHODS

### Cell culture

Human lung carcinoma cells A549, colon carcinoma RKO, and breast carcinoma T47D and MCF-7 cells (all from ATCC) were cultured in DMEM with 10% FCS (Biochrome), at 37°C in humidified air containing 5% CO_2_. Cells were treated with etoposide (25 μM), paclitaxel (12.5 or 25 nM), UV irradiation (12 J/m^2^), and camptothecin (2 μM) for different time periods depending on experimental settings.

### Plasmids and transfections

The full-length S100P cDNA was subcloned into the pcDNA 3.1 plasmid (Invitrogen) from the pSG5C-S100P vector [20]. Construction of the pGEX-3X-S100P plasmid was described elsewhere [49]. F15A mutation was inserted by PCR amplification of the pGEX-3X-S100P plasmid with the primers listed in the Supplementary Table S1. Transfections were performed with TurboFect reagent (ThermoScientific) with 2 μg of the plasmid DNA added to cells at 60-80% confluence in 35-mm Petri dishes. Transiently transfected cells were analyzed 24-72 h post-transfection. Stable transfectants were obtained by selection with 800 μg/ml G418, and expansion of surviving cells either as a whole population or as clonal cell lines.

### S100P silencing

In order to transiently suppress the S100P expression, MCF-7 cells were transfected with 10 nM S100P siRNA (h): sc- 61488 (Santa Cruz Biotechnology) using the Gene Silencer siRNA Transfection Reagent (Genlantis) according to the manufacturer's instructions. Ten nanomolar Silencer Negative Control siRNA (Applied Biosystems) was used as control. 48 h after transfection, the cells were treated with PTX and UV and incubated for additional 24 hours. The RNA was isolated and analyzed by real-time quantitative PCR as described above.

For the stable S100P suppression, the MCF-7 cell line was transfected by pRNATin-1.2/Hygro/shRNA scr (negative control) and pRNATin-1.2/Hygro/sh-S100P, respectively, and selected in Hygromycin B.

Following pairs of oligonucleotides were cloned into the BamHI/HindIII-digested and dephosphorylated pRNATin-1.2/Hygro: siS100P top strand 5′-GATCCGTG CCGTGGATAAATTGCTCAATT

CGTGGATAAATTGCTCAATTGATATCCGTTGAGCAATTTATCCACGGCATT TTTTGGAAA-3′ and bottom strand 5′-AGCTT TTCCAAAAAATGCCGTGGATAAATTGCTCAACGGATATCAATTGAGCAATTTATCCACGGCACG-3′); control scr top strand 5′-GATCCGAGATCCGTATAGTGTACCTTATTGATATCCGTAAGGTACACTATACGGA TCTTTTTTT GGAAA-3′ and bottom strand 5′-AGCTTTTCCAAAAAAAGATCCGTATAGTGTACCTTACGGATATCAAAAGGTACACTATACGGATCTCG-3′. The selected cells were expanded and analyzed for senescence and colony formation as described below.

### Antibodies

Primary antibodies, including the mouse monoclonal antibody (MAb) DO-1 specific for p53 [50], the mouse MAbs 2A9 and 2A10 specific for HDM2 [51], and the rabbit polyclonal antibody CM-1 specific for p53 [52, 53], were diluted 1:1000 and used either for immunoblotting or for proximity ligation assay (PLA); the goat polyclonal anti-human actin C-11 antibody in 1:1000 was used for loading control (Santa Cruz Biotech); and the mouse anti-S100P MAb in undiluted hybridoma medium [49,54] was used for immunoblotting and PLA. HRP-conjugated goat anti-mouse IgG and HRP-conjugated rabbit anti-goat IgG (both from DAKO) were diluted 1:5000 and used as secondary antibodies.

### GST pull-down

The recombinant proteins GST-wtS100P, GST-mutS100P (containing F15A mutation), and GST in supernatants from bacterial lysates were immobilized on Glutathione-S-Sepharose 4B beads. Washed beads were incubated overnight with pre-cleared lysates of RKO and T47D cells containing either 0.7 mM CaCl_2_ or 0.7 mM EGTA. After repeated washing, the samples were resolved in SDS-PAGE and analyzed by immunoblotting using DO-1 and 2A9 antibodies [50, 51].

### Immunoblotting

Cells grown in confluent monolayers were rinsed twice with cold PBS, re-suspended in lysis buffer (1% Triton X-100; 50 mM Tris pH 7,5; 150 mM NaCl; 0.5% Nonidet P-40) containing the cocktail of protease inhibitors (Roche) and cleared by centrifugation. Proteins were quantified using the BCA protein assay (Pierce). Protein extracts (100 μg/lane) were resolved in 10% SDS-PAGE and transferred to a PVDF membrane (Macherey-Nagel). The proteins were detected with appropriate antibodies and visualized using an enhanced chemiluminescence kit (GE Healthcare Bio-Science). Far Western blotting was performed as described in the legend to the Supplemental Figure S1 according to [55].

### xCELLigence real-time cell assay (RTCA)

RTCA was used for monitoring of cell proliferation and viability in real-time. Experiments were set up in E-Plates 16 (Roche). Background impedance was measured in 100 μl of cell culture medium/well. RKO-empty pcDNA 3.1 and RKO-S100P cells were plated at 7×10^3^ cells/well (adjusted to the final volume of 200 μl). The impedance was recorded in 15 min intervals for 24 h. After administration of 25 nM paclitaxel, the impedance was recorded in 5 min intervals for additional 36 h in quadruplicates. Recorded values were presented as Cell Index (CI) calculated as a relative change in the electrical impedance.

### Proximity ligation assay (PLA)

PLA was used for in situ detection of the protein-protein interactions [22]. Cells were seeded on glass coverslips, allowed to attach before treatment, and cultured for 24 h. Then they were fixed with 4% paraformaldehyde, permeabilized with 0.1 % Triton X, and assayed in a humid chamber at 37°C (Olink Bioscience). Signal representing the interaction between the proteins of interest was analyzed using the Zeiss LSM 510 Meta confocal microscope.

### Flow cytometric analysis of cell viability (FACS)

Treated cells were harvested (at 1×10^6^ cells/sample), washed in PBS, labeled and analyzed by flow cytometer FACSCanto™ II (Beckton Dickinson) equipped with 488 nm laser used for dye excitation. Labeling of viable cells was performed in 300 μl of PBS with 10 nM fluorescein diacetate (FDA) for 25 minutes at room temperature in the dark followed by propidium iodide (PI) at final concentration of 5 μg/ml. Emitted fluorescence was collected using the 530/30 filter for FDA and 585/42 filter for PI. Viable populations showed positive staining for FDA and negative staining for PI. Samples for subG1 analysis were treated with 0.05% Triton X100 in 300 μl of PBS for 25 minutes at 37°C and stained with 50 μg/ml PI. Emitted PI fluorescence was collected with the 585/42 filter. Data for the assessment of cell viability and SubG1% were analyzed with the FCS Express version 4.0 (De Novo Software).

### Profiling of apoptosis-related proteins

Expression profile of apoptosis-related proteins was analyzed using a Human apoptosis array kit (R&D Systems). The membranes with immobilized antibodies were incubated with the lysates of RKO cells transiently transfected with empty pcDNA 3.1 or pcDNA 3.1-FL-S100P, untreated or treated with paclitaxel (25 nM for 4 h), etoposide (25 μM for 6 h), or camptothecin (2 μM for 4h). Washed membranes were developed by ECL and exposed to X-ray films. The films were scanned and pixel density was evaluated by quantifying the mean duplicate spots densities from two separate experiments. For the quantification, the spot volume was determined, corrected for background and values were expressed as % difference between S100P-expressing versus control cells.

### Real-time quantitative PCR (qPCR)

Total RNA was isolated using Instapure solution (Eurogentech). Reverse transcription of RNA was performed with the High-Capacity cDNA Reverse Transcription kit (Applied Biosystems). Amplification was performed in the Stratagene Mx 3005P thermal cycling block (Agilent Technologies). PCR was carried out in 20-μl volumes using Maxima Syber Green PCR Master Mix (Fermentas) for 10 min at 95°C for initial denaturation followed by 40 cycles of 95°C for 15 s and 60°C for 1 min, using primers listed in the Supplementary Table S1. Sample Ct values were normalized to actin. Relative expression was calculated using the ΔΔCt method. Amplifications were performed in triplicates in 3-5 independent experiments.

### Senescence-associated β-Galactosidase assay

SA-β-Gal activity was detected by the Senescence β-Galactosidase Staining Kit (Cell Signalling Technology). Transfected RKO cells were seeded in 30-mm Petri dishes. Following the drug treatment for 72 h, the cells were washed, fixed and stained for 24 h at 37°C in absence of CO_2_. The cells were viewed using the phase contrast microscope. Senescent cells were recognized according to blue staining.

### Colony outgrowth assay

The cells were plated at 10% density in 6 cm dishes and exposed to camptothecin or paclitaxel (at 100 nM and 25 nM concentrations) in triplicates for three days. On day four, the cells were recovered in fresh media for additional 14 days with the growth medium replacement every 4 days. After extensive washing with PBS and fixation with 4% paraformaldehyde, the remaining colonies were stained with 0.5% Coomassie blue or Giemsa for 5 min at room temperature, extensively washed with PBS, dried and photographed.

### Statistical analysis

Results were analyzed by two-tailed unpaired t test (Student's test), and p<0.05 was considered significant.

## SUPPLEMENTARY FIGURES AND TABLE


