# Supplementary material for: Cancer-associated S100P protein binds and inactivates p53, permits therapy-induced senescence and supports chemoresistance
Source: Oncotarget. 2016 Mar 9;7(16):22508–22. doi: 10.18632/oncotarget.7999 (PMC5008377; doi:10.18632/oncotarget.7999)
Supplement: Supplementary file 1 [file oncotarget-07-22508-s001.pdf]

SUPPLEMENTARY FIGURES AND TABLE

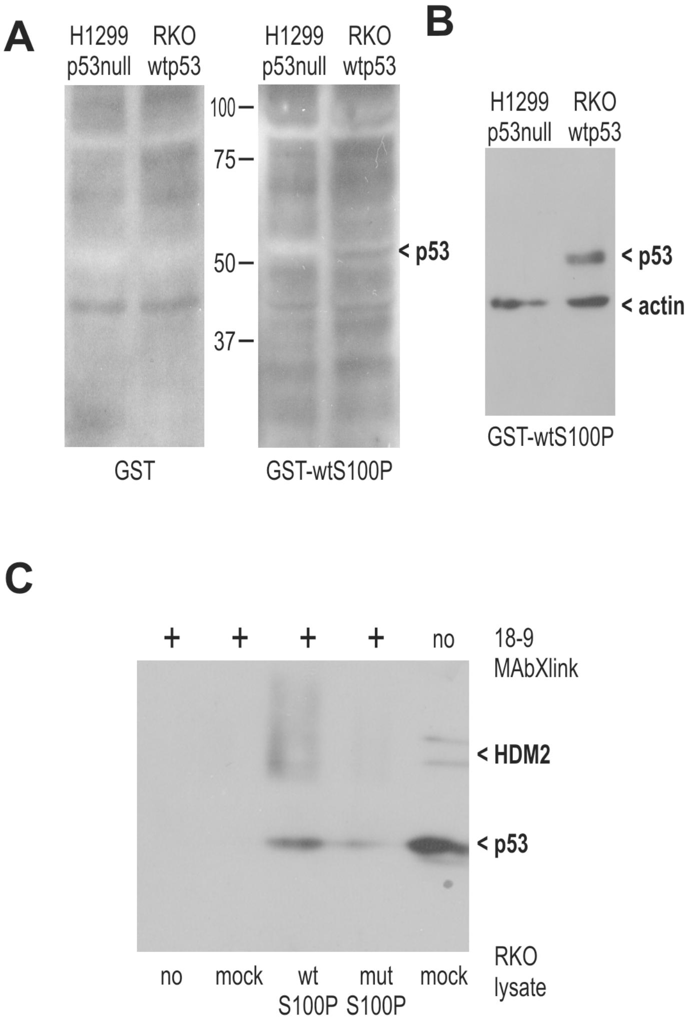

Supplementary Figure S1: Analysis of the S100P interaction with p53 and/or HDM2 using Far Western blotting and co-immunoprecipitation. A. Far Western analysis.Prey proteins in the lysates of p53-null NCI-H1299 and wild-type p53 containing UV-treated RKO cells were run on SDS-PAGE and blotted on the PVDF membrane, then renaturated using guanidine hydrochloride according to [55]. Then the membrane was blocked and incubated with bait proteins, namely GST and GST-wtS100P. Binding of the bait proteins to the blot was detected using the anti-S100P Mab 18-9. Band related to p53 is visible only in cells expressing the p53-competent RKO cells incubated with the GST-wtS100P fusion protein. **B.** Corresponding immunoblot incubated with antibodies directed to p53 and actin. **C.** Co-immunoprecipitation of p53 and HDM2 proteins using the S100P-specific antibody. Purified MAb 18-9 was bound to Protein G immobilized on 4% agarose beads, washed and cross-linked as described in https://www3.nd.edu/~clarklab/protocols/immunoassays/immunoppt.pdf. Quenched and washed beads were then incubated overnight with pre-cleared lysates of RKO, RKO-FL-S100P and RKO-mutS100P transfected cells, one aliquot of AbXlink was spared as control. After repeated washing, the samples were resolved on SDS-PAGE and analyzed by immunoblotting using DO-1 and 2A9 antibodies against p53 and HDM2 proteins.

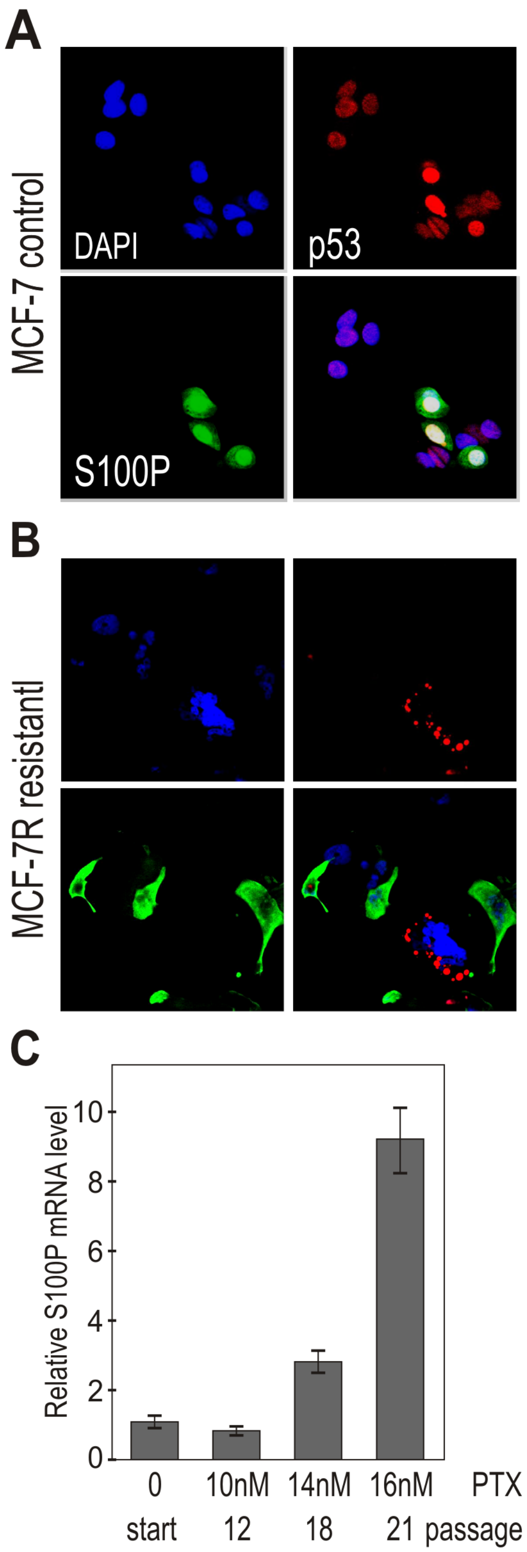

Supplementary Figure S2: S100P expression in paclitaxel-resistant MCF-7R cells. MCF-7 cell line with acquired resistance to paclitaxel was generated by growth in incrementally increasing PTX concentrations with successive passages (starting with 1 nM paclitaxel). The resulting cell line with PTX resistance, designated MCF-7R, was maintained at 16 nM paclitaxel concentration with passage number exceeding 21. Parental MCF-7 cells **A.** and PTX-resistant MCF-7R cells **B.** were stained for S100P (green), p53 (red) and nuclei (blue) and subjected to immunofluorescence analysis using the confocal microscopy. **C.** Q-PCR analysis of S100P expression during the development of chemoresistant MCF-7R cells. RNA was isolated from cells growing in 10 nM PTX (passage 12, week 6), 14 nM PTX (passage 18, week 9) and 16 nM PTX (passage 21, week 12).

Supplementary Table S1: List of primers used for cloning and Q-PCR

Gene	Sense primer (5'-3')	Antisense primer (5'-3')
mutS100P(F15A)	CATAGACGTCGCTTCCCGATATTC	CGAATATCGGGAAGCGACGTCTATG
Actin	TCCTCCCTGGAGAAGAGCTA	ACATCTGCTGGAAGGTGGAC
Bax	GGACGAACTGGACAGTAACATGG	GCAAAGTAGAAAAGGGCGACAAC
HDM2	GCAAATGTGCAATACCAACATGTC	GCCAAACAAATCTCCTAGAAGATC
$p21^{WAF}$	AGTCCTGGAAGCGCGAGGGC	GGTGGCTTTAAATAGTATTTCATAAAA
TP53	TCCCTTCCCAGAAAACCTACCA	TCATAGGGCACCACCACACTC
S100P	AAGGGGGAGCTCAAGGTGCTGA	ATCTGTGACATCTCCAGGGCATC
